# Acerola and Its By-Products as Sources of Bioactive Compounds: Phytochemical Profile and Biological Effects in Experimental and Clinical Studies

**DOI:** 10.3390/molecules31111792

**Published:** 2026-05-23

**Authors:** Jailane de Souza Aquino, Alana Natalícia Vasconcelos de Araújo, Januse Míllia Dantas de Araújo, Luana Clementino Santos, Jordania Candice Costa Silva, Kamila Sabino Batista, Lucas Rannier Ribeiro Antonino Carvalho

**Affiliations:** 1 Postgraduate Program in Nutrition Sciences, Federal University of Paraíba, João Pessoa 58051-900, Paraíba, Brazil; jailane.aquino@academico.ufpb.br (J.d.S.A.); allana1araujo@gmail.com (A.N.V.d.A.); januse.millia@academico.ufpb.br (J.M.D.d.A.); 2 Postgraduate Program in Food Science and Technology, Federal University of Paraíba, João Pessoa 58051-900, Paraíba, Brazil; luana.clementino@academico.ufpb.br (L.C.S.); jordania.candice@academico.ufpb.br (J.C.C.S.); 3 Experimental Nutrition Laboratory—LANEX, Department of Nutrition, Federal University of Paraíba, João Pessoa 58051-900, Paraíba, Brazil; 4 Secretariat for Health and Environmental Surveillance, Ministry of Health, Brasília 70719-040, Federal District, Brazil; kamilasabino00@gmail.com; 5 Department of Physiology and Pharmacology, Karolinska Institutet, Biomedicum, Solnavägen 9, SE-171 65 Solna, Sweden

**Keywords:** acerola, *Malpighia emarginata*, fruit by-products, phytochemical composition, animal studies, clinical trials, antioxidant activity, metabolic effects

## Abstract

Acerola (*Malpighia emarginata* DC.) is one of the richest natural sources of vitamin C and an important source of phenolic compounds, carotenoids, and bioactive polysaccharides. Although the fruit can be consumed fresh, it is more commonly processed into juices and frozen pulp, generating substantial amounts of by-products (pomace, peels, and seeds), corresponding to approximately 20–60% of the fruit biomass, with high phytochemical content. These fractions represent underutilized sources of bioactive compounds. This narrative review, supported by a structured literature search, integrates evidence on the phytochemical composition of acerola pulp and its by-products and relates these profiles to biological effects in experimental and human studies, focusing on compound characterization, composition–function relationships, and underlying mechanisms. Key compounds, including ascorbic acid, hydroxycinnamic acids, flavonoids, and polysaccharides, are associated with the modulation of redox homeostasis, inflammatory signaling, and lipid metabolism, particularly under high-fat dietary conditions. Human evidence remains limited but suggests matrix-dependent effects on vitamin C bioavailability and selected cardiometabolic markers. Overall, the evidence is constrained by methodological heterogeneity, limited clinical data, and insufficient characterization of bioactive fractions. Future research should prioritize detailed phytochemical profiling, dose–response relationships, bioavailability assessment, and well-controlled clinical trials incorporating molecular biomarkers, supporting the development of acerola-derived matrices as functional and bioactive-rich ingredients.

## 1. Introduction

The growing demand for sustainable food systems has increased interest in the valorization of fruit by-products as alternative sources of bioactive compounds with potential health benefits [1,2,3]. Large volumes of by-products are generated during fruit processing, particularly in juice and pulp industries, representing both an environmental challenge and an opportunity for the recovery of high-value phytochemicals [2,4,5]. Tropical fruits are especially relevant in this context due to their chemical diversity and high concentrations of bioactive compounds [6,7].

Acerola (*Malpighia emarginata* DC.), also known as Barbados cherry or West Indian cherry, is a perennial species native to the Caribbean Basin and northern South America and is currently cultivated in tropical and subtropical regions [8]. Brazil is the leading producer, with approximately 5753 hectares under cultivation and an annual production of 60,000–61,000 tons, largely concentrated in the Northeast region [9]. This production supports an industrial chain focused on frozen pulp, juices, concentrates, and nutraceutical ingredients. During processing, a substantial proportion of the fruit biomass estimated at 20–60% is converted into by-products, mainly pomace, peels and seeds [4,9].

These by-products may contain equal or higher levels of phenolic compounds, carotenoids, and residual ascorbic acid than the edible pulp [4,5]. Acerola is also a rich source of vitamin C and contains flavonoids and hydroxycinnamic acids associated with antioxidant and anti-inflammatory effects [4,10].

Both acerola and its by-products have been increasingly explored in food and nutraceutical applications [11,12,13,14]. The pulp has been incorporated into fermented and dairy products with good sensory acceptance [11], while processing strategies, particularly drying, have improved stability and expanded its use in various food formulations [12]. Acerola by-products have also been investigated as natural antioxidant ingredients in functional foods and dietary supplements, and immature fruits are widely used as industrial sources of ascorbic acid [13,14].

Phenolic extracts from acerola by-products have been applied to the development of biodegradable films and coatings, contributing to improved preservation and extended shelf life [15,16,17,18]. Technological approaches have facilitated their stabilization and incorporation into complex matrices [12,19,20], while emerging evidence suggests their potential to enhance the gastrointestinal survival of probiotic strains [21]. More recently, acerola-derived matrices have been explored in advanced delivery systems, including naturally occurring nanovesicles capable of protecting bioactive compounds during gastrointestinal transit [22]. Together, these findings highlight the technological versatility of acerola and its by-products and their relevance for nutraceutical and functional applications.

However, evidence regarding their biological effects remains uneven and is largely derived from preclinical models, with relatively few human studies [8,14,23]. The frequent use of simplified experimental designs, crude extracts, and antioxidant-focused assays limits mechanistic insight and hampers the identification of molecular targets and dose–response relationships [1,13,14,23,24,25,26,27,28].

In parallel, most reviews on acerola have addressed composition, applications, or selected biological activities as separate topics [1,4,8,14,23], leaving the integration of phytochemical composition with biological effects across experimental settings insufficiently explored. The application of omics-based approaches also remains limited [29], and the role of matrix complexity (whole matrices versus isolated compounds) in shaping biological responses has not been comprehensively evaluated [27,30].

Thus, this narrative review examines the phytochemical composition of acerola pulp and its by-products and relates these profiles to their biological effects in experimental and clinical studies. To address the identified gap, this review provides an integrative analysis linking phytochemical composition to biological effects across different experimental settings, an approach that has not been systematically explored in previous studies. Specifically, it focuses on three complementary aspects: (i) the characterization of bioactive compounds; (ii) the integration of compositional data with reported biological effects across different experimental models; and (iii) the identification of underlying mechanisms linking specific compounds to physiological responses. By structuring the analysis in this way, the review provides an integrated perspective and outlines directions for future research, supporting the development of acerola-derived matrices as functional and bioactive-rich ingredients.

## 2. Literature Search and Study Selection

This study was conducted as a narrative review supported by a structured literature search. A comprehensive search was performed in the Embase, PubMed/MEDLINE, Scopus, and Web of Science Core Collection databases to identify studies on acerola, its bioactive compounds, and related biological effects. The search was performed in February 2026 using a combination of controlled vocabulary (Emtree and MeSH terms) and free-text terms, adapted to the syntax of each database.

The search strategy included terms related to acerola (e.g., “acerola”, “*Malpighia emarginata*”, “Barbados cherry”, “West Indian cherry”, and “Antilles cherry”), combined with terms associated with bioactive compounds and antioxidant properties (e.g., “antioxidant*”, “phenolic*”, “polyphenol*”, “flavonoid*”, “ascorbic acid”, “vitamin C”, “extract*”, and “phytochemical*”), as well as terms related to biological activity and experimental models (e.g., “effect*”, “activ*”, “bioactiv*”, “metabol*”, “animal*”, “in vivo”, “human”, and “experiment*”). Boolean operators (AND and OR) were used to combine these terms. In addition, citation searching was performed to identify relevant studies not captured in the database search.

**Figure 1:**
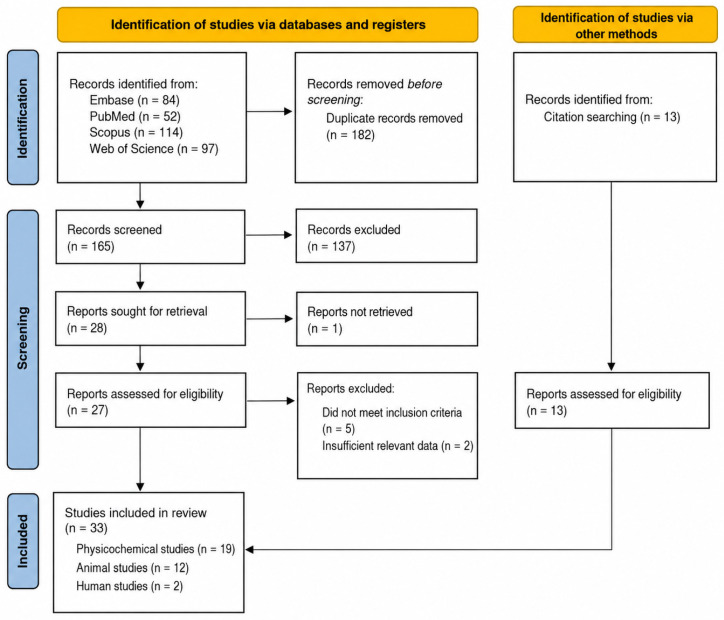
The PRSIMA 2020 flow diagram used to select the articles.

Study selection was performed according to predefined inclusion and exclusion criteria. Only original articles published in English between January 2016 and February 2026 were considered. Reviews, editorials, conference abstracts, and studies not aligned with the scope of this review were excluded. Screening was performed based on titles and abstracts, followed by full-text assessment. A total of 33 studies met the inclusion criteria and were included in this review. The study selection process was summarized using the PRISMA 2020 flow diagram framework (Figure 1). As this study was conducted as a narrative review supported by a structured literature search, no formal risk of bias assessment was performed.

## 3. Phytochemical Profile and Biological Effects of Acerola and Its By-Products

Acerola is characterized by pronounced genotypic heterogeneity, resulting in significant variation in fruit morphology, pulp yield, soluble solids content, and, importantly, the secondary metabolites profiles [10,13,31]. Internationally, acerola cultivars are less standardized than those developed in Brazil and are often described as local or regional selections [32]. In the Caribbean, Central America, and tropical regions of the United States (such as Florida and Hawaii), common types include Barbados or West Indian, as well as Florida Sweet and Tropical Sweet. These cultivars are typically selected for traits like yield, fruit size, and lower acidity for fresh consumption. However, many are not officially registered, which limits standardization and comparability across studies, reflecting the high genetic variability of the species [31,32,33].

Commercial cultivars cultivated in Brazil, such as ‘Okinawa’, ‘Sertaneja’, ‘Flor Branca’, ‘Junko’, and ‘Costa Rica’, and internationally disseminated genotypes including ‘Florida Sweet’ and ‘B-17’, represent distinct biochemical phenotypes shaped by breeding selection and agroecological adaptation [34]. More recently, regionally adapted breeding initiatives have generated improved genotypes such as ‘Rubi-PB’, developed through collaboration between researchers from the Empresa Paraibana de Pesquisa, Extensão e Regularização Fundiária (EMPAER-PB) and the Federal University of Paraíba (UFPB), selected for yield stability, elevated soluble solids (°Brix), and high ascorbic acid concentration under semi-arid stress conditions [35].

Importantly, comparative analyses demonstrate that genotype and processing parameters modulate the relative abundance and structural diversity of phenolic compounds, carotenoids, and residual ascorbic acid, thereby influencing both quantitative bioactive density and qualitative phytochemical complexity [36].

**Figure 2:**
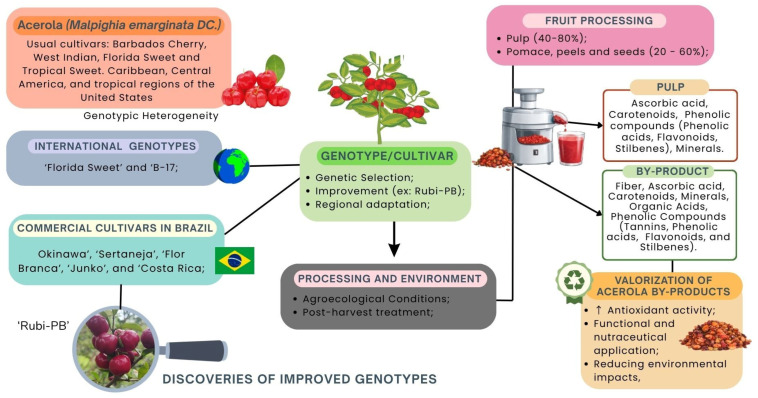
Processing flow of acerola (*Malpighia emarginata* DC.) and generation of value-added by-products. The diagram shows acerola processing and the resulting by-products (pomace, peels, and seeds), highlighting the phytochemical composition of pulp and by-product fractions and their potential for functional and nutraceutical applications. Arrow (↑) = indicates increase.

Genotypic variability and processing conditions are recognized determinants of the qualitative and quantitative phytochemical composition of acerola matrices, with direct implications for biological activity [31]. An integrated framework linking genotypic variability, processing conditions, and the distribution of bioactive compounds in pulp and by-products is presented in Figure 2. Nevertheless, cultivar identity is frequently not reported in experimental animal and human studies, thereby limiting robust integration between composition and biological function [4,31]. The following sections critically synthesize the available phytochemical evidence within this framework, emphasizing genotype traceability as a key factor for strengthening translational interpretation.

### 3.1. Proximate Composition and Phytochemical Profile of Acerola Pulp

The phytochemical profile of acerola pulp is characterized by a high content of bioactive compounds. On a dry weight basis, high concentrations of total phenolic compounds (2069.55 mg/100 g), ascorbic acid (7.94–36.36 mg/100 g), and total anthocyanins (2.24 mg/100 g) have been reported, with cyanidin-3-rhamnoside identified as the predominant pigment [37,38]. In addition, the pulp contains 3.22 µg/g of total chlorophyll and 29.71–79.2 µg/g of total carotenoids [37,38]. Previous research on acerola pulp cultivated in Brazil (cultivar not reported in the original study) identified hydroxycinnamic acids (chlorogenic and gallic acids), flavan-3-ols (catechin, procyanidin B1, and epigallocatechin gallate), and flavonols (kaempferol 3-glycoside and quercetin 3-glycoside) as the predominant quantified polyphenols [37]. The phytochemical profile and proximate composition of acerola pulp are presented in Table 1.

The quantification of ascorbic acid, total chlorophyll, flavonoids, and carotenoids in studies involving acerola pulp was performed by spectrophotometry, using specific methodologies for each class of compound based on absorbance at characteristic wavelengths [13,39]. The total phenolic content was determined using the Folin–Ciocalteu colorimetric method, which is widely employed to estimate the overall phenolic content in plant matrices [13]. Additionally, the phenolic profile was identified and quantified by high-performance liquid chromatography coupled with a UV/Vis detector (HPLC-UV/Vis), enabling the separation, identification, and quantification of the different compounds present in the samples [37].

**Table 1:** Proximate composition and main bioactive compounds of acerola pulp *.

Parameter (g/100 g)	Mean Value	Reference
Moisture	93.49	[38]
	91.20	[40]
Proteins	0.04	[38]
	1.08	[40]
Lipids	0.09	[38]
	0.53	[40]
Ash	0.60	[38]
	0.31	[40]
	0.36	[37]
Carbohydrates	5.45	[38]
	6.28	[40]
Soluble fibers	0.24	[40]
Insoluble fibers	1.14	[40]
Bioactive compounds		
Ascorbic acid (mg/100 g)	36.36	[37]
	7.94	[38]
Lycopene (mg/100 g)	0.19	[37]
Anthocyanins (mg/100 g)	2.24	[37]
Total chlorophyll (μg/g)	3.22	[38]
Carotenoids (μg/g)		
Trans-β-carotene	11.70	[40]
Trans-α-carotene	1.24	
β-cryptoxanthin	1.92	
9-cis-βC	0.73	
13-cis-βC	1.44	
15-cis-βC	1.19	
Lutein	1.00	
Total carotenoids	79.20	[37]
	29.71	[38]
Phenolic compounds (mg/100 g)		
Gallic acid	2.20	[37]
Syringic acid	0.03	
Caftaric acid	0.11	
Chlorogenic acid	0.15	
Caffeic acid	0.06	
p-Coumaric acid	0.03	
Procyanidin B1	0.40	
Procyanidin B2	0.17	
Epigallocatechin gallate	0.32	
Epicatechin	0.07	
Epicatechin gallate	0.08	
Catechin	1.14	
Kaempferol 3-glicoside	1.39	
Rutin	0.18	
Quercetin 3-Glycoside	0.66	
Isorhamnetin	0.24	
Hesperidin	0.30	
Trans-resveratrol	0.10	
Total flavonoids (mg/100 g)	12.64	[37]
	3.74	[38]
Total phenolic compounds (mg/100 g)	2069.55	[37]
	6.12	[38]
Minerals (mg/100 g)		
Iron	0.80	[41]
Zinc	0.08	
Manganese	0.24	
Copper	0.17	
Sodium	35.13	
Aluminum	0.93	
Boron	0.11	

* Values are presented as means to ensure consistency across studies, as standard deviations were not consistently reported in all the original sources.

The differences observed in bioactive compound values between studies can be attributed to multiple methodological factors, including variations in extraction procedures (e.g., solvent type, time, temperature, and processing conditions), as well as differences in the units or bases used to express the results (fresh weight basis, dry weight basis, or gallic acid equivalents). In addition, variability in the sample matrix, fruit ripening stage, cultivar/variety, and cultivation conditions may also contribute to the observed differences. Collectively, these factors limit the direct comparability between studies [17,37].

Acerola pulp also contains carotenoids, which, together with ascorbic acid and phenolic profile, may contribute to its high antioxidant activity [36,42,43]. A comparative assessment indicates that, although acerola contains relevant levels of carotenoids, it cannot be classified among the most concentrated dietary sources when compared to well-established carotenoid-rich matrices such as mango, papaya and tomato [44,45,46]. Nevertheless, its carotenoid, ascorbic acid and phenolic contents are comparable to, and in some cases higher than those of widely consumed fruits such as apple, guava, orange and lemon, depending on genotype, ripening stage, and processing conditions [31,37,47,48].

Chemical characterization studies have demonstrated the presence of tocopherols (vitamin E), phytosterols such as β-sitosterol, campesterol, and stigmasterol, as well as saponins and trace levels of alkaloids, compounds that contribute to the fruit’s antioxidant and anti-inflammatory potential [6,23]. Volatile compounds, including terpenes with possible antimicrobial and antioxidant activity, have also been identified in the acerola matrix [49].

Several studies have demonstrated that acerola pulp (with the cultivar not reported in the original study) exhibits high antioxidant capacity, as measured by standard free radical scavenging and reducing power assays. In addition to recent data reporting antioxidant activity of approximately 6.17 μmol Trolox equivalents/g by the DPPH assay and approximately 3.19 mM TEAC/g by the ABTS assay, broader investigations have shown expressive values for ORAC and other antioxidant capacity methods [38]. In a comparative study, acerola pulp (cultivar not reported in the original study) exhibited higher antioxidant capacity, as measured by ABTS (21.99 µmol Fe^2+^/g), DPPH (11.14 µmol TE/g), and FRAP (6.71 µmol TE/g) assays, with values higher than those observed for guava [37].

In different analyses, the ORAC method applied to acerola fruits at different ripening stages from two distinct regions in the United States (Davie and Vero Beach, Florida) showed values ranging approximately from 36 to 79 mM Trolox equivalents/kg of fresh weight basis, depending on geographic origin and maturity stage [49]. Phenolic compounds were responsible for approximately 7.1–36.5% of the total antioxidant capacity measured by the ORAC assay, while ascorbic acid contributed between 18% and 39% of the overall antioxidant activity [49]. Regardless of geographic origin, fruits at earlier ripening stages consistently exhibited higher antioxidant capacity, as measured by the ORAC method, compared to fully ripe fruits. This trend is mainly attributed to the higher concentration of phenolic compounds in unripe or semi-ripe stages, which significantly contribute to total antioxidant activity [49]. As ripening progresses, a general reduction in phenolic content and ascorbic acid levels is observed, leading to a decrease in overall antioxidant capacity [49]. Nevertheless, these findings reflect a high oxygen radical scavenging capacity, indicating effective antioxidant activity at the molecular level [49].

Evidence has demonstrated that extracts from unripe acerola pulp (with the cultivar not reported in the original study) exhibited higher antioxidant capacity and reduced power compared to those from ripe fruit. In the current analysis, ascorbic acid contributed between 59.12% and 98.12% of the antioxidant activity, with the lowest contribution observed in ripe acerola and the highest in unripe acerola [50]. These results help explain the elevated reducing power observed in unripe samples and indicate that this method should not be used as a proxy for total phenolic content, since ascorbic acid is not a phenolic compound [50].

### 3.2. Proximate Composition and Phytochemical Profile of Acerola By-Products

During industrial processing of acerola mainly to produce juice and frozen pulp, substantial amounts of by-products, including pomace, peels, and seeds, are generated [4,5]. The extensive processing of acerola has raised environmental concerns, particularly regarding waste management. These by-products represent, on average, 40% of the total fruit volume, indicating a substantial loss of biomass that could otherwise be recovered and valorized in the development of value-added products [1].

The by-products generated during the processing of acerola have been characterized as nutritionally and functionally valuable matrices. According to da Silva et al. [17], acerola pulp by-product, on a dry weight basis, contains 34.2 ± 0.4 g/100 g of crude fiber and 42.3 mg/100 g of anthocyanins, expressed on a wet weight basis. Complementarily, Batista et al. [30] identified even higher concentrations of specific bioactives, reporting 5366.44 µg/g of total phenolic compounds (536.6 mg/100 g) and 2022.06 µg/g ascorbic acid. Their work also quantified individual flavonoids, including quercetin (250.80 µg/g) and rutin (22.46 µg/g), as well as total carotenoids (4.99 mg/100 g).

The studies involving acerola by-products (cultivar not reported in the original study) presented in Table 2 characterized the profile of bioactive compounds using different analytical methodologies, depending on the compound class evaluated. Anthocyanin determination was performed on a wet basis after acidified aqueous extraction, followed by quantification using UV–Vis spectrophotometry at 520 and 700 nm [17]. Chlorophyll content was also determined by spectrophotometric readings at wavelengths of 646 [50] and 663 nm [51]. Total carotenoids were also determined by UV–Vis spectrophotometry at 450, 470 and 645 nm [30,51,52]. For ascorbic acid, different approaches were employed across the studies, including organic solvent extraction followed by HPLC-DAD analysis [17], UV-Vis spectrophotometry at 518 nm [30], and the titrimetric method [52]. Total phenolic content was determined by the Folin–Ciocalteu assay at different absorbances (UV-Vis, 725 nm) [17] and (UV-Vis, 760 nm) [51,53]. Additionally, the phenolic and organic acids profile was characterized using organic solvent extraction and high-performance liquid chromatography coupled with a DAD detector (HPLC-DAD), enabling the identification and quantification of individual compounds [30].

Liquid chromatographic (HPLC-DAD) profiling further identified phenolic compounds such as hesperetin, naringenin, and phenolic acids, as well as fructooligosaccharides (kestose and nystose), reinforcing the multifunctional nature of these matrices [30,54]. The phytochemical composition and physicochemical characteristics of acerola by-products are summarized in Table 2.

The substantial variability reported across characterization studies represents a key limitation for direct comparisons between matrices, as it is influenced by factors such as genotype, ripening stage, processing conditions, and the interval between harvest and analysis. Furthermore, differences in analytical methodologies and reporting units (e.g., fresh vs. dry weight) compromise cross-study comparability and may introduce bias [55].

Importantly, this variability is compound-dependent. Ascorbic acid shows particularly high variability due to its high susceptibility to oxidation and differences in extraction conditions, whereas carotenoids are relatively more stable, although still influenced by processing, light exposure, and solvent systems [44,56]. Anthocyanins, in turn, are highly affected by pH, temperature, and extraction protocols, contributing to inconsistencies across studies. Phenolic compounds also exhibit considerable variation, largely due to differences in extraction solvents, hydrolysis steps, analytical methods, and their structural diversity [46,57]. In addition, dietary fiber content may vary depending on the type of by-product analyzed (e.g., peel, seed, or pomace) and the processing conditions applied, particularly those affecting the proportion of soluble and insoluble fractions [56].

These limitations are further exacerbated by the lack of standardization in data reporting, including differences between fresh and dry weight bases, expression per edible portion or per extract, and the use of distinct normalization criteria (e.g., per 100 g, per gram, or per serving size). Moreover, variations in moisture content and sample preparation can significantly influence reported concentrations, leading to over- or underestimation of bioactive compound levels and reducing the reliability of direct comparisons and meta-analytical interpretations [55].

Focusing on acerola, fruit by-products exhibit significant variability in the contents of phenolic compounds and dietary fiber. While grape pomace is recognized as one of the richest matrices, presenting higher levels of these compounds than acerola, by-products from banana, pineapple, and mango generally show low to moderate levels compared to acerola [2]. Acerola residues present relatively high levels of ascorbic acid, comparable to citrus and apple by-products, although generally lower than those derived from grapes. Regarding carotenoids, acerola by-products exhibit higher levels than those of fruits such as apple and strawberry, but lower than well-established sources such as mango and carrot [2,57,58,59].

Acerola by-products, which comprise residual pulp and peel fractions, are particularly rich in flavonoids and phenolic acids, exhibiting strong antioxidant activity in chemical and biological assays [54]. Peels have been reported to contain concentrated phenolic fractions, while seeds, although less explored, have shown promising levels of tannins and other phenolic compounds with antioxidant potential [31]. These findings reinforce the concept that acerola by-products represent underutilized matrices with relevant phytochemical and biological value.

Importantly, the antioxidant activity of acerola cannot be attributed solely to vitamin C. Several studies have demonstrated synergistic effects between ascorbic acid and polyphenols, resulting in enhanced radical scavenging capacity and improved biological activity when compared to isolated compounds [6,7]. This complex phytochemical matrix supports the classification of acerola as a functional fruit with potential applications beyond the present total phenolic contents comparable to or higher than those found in the edible pulp, highlighting its potential as an alternative source of phytochemicals [38,39].

Acerola by-products have been widely investigated for their antioxidant capacity and activity, due to the high concentration of bioactive compounds that remain after industrial processing. Studies have shown that these by-products contain substantial levels of total phenolic compounds, with concentrations that may be comparable to or even exceed those found in the pulp [1,17].

Antioxidant activity is commonly assessed using assays such as DPPH, ABTS, FRAP, and ORAC. Across studies, acerola by-products consistently exhibit high antioxidant capacity, although results vary depending on the assay and experimental conditions. ABTS values have been reported within a relatively narrow range (46.7–48.5 µmol Trolox equivalents (TE)/g dry weight), whereas FRAP values show greater dispersion (93.0–273.8 µmol TE/g dry weight basis), indicating variability in reducing power across samples [17]. Similarly, DPPH results reported values (38.17 EC_50_ μg/mL extract) indicate strong radical scavenging capacity, while ORAC values (7.86 ± 0.06 EC_50_ μg/mL extract) confirm the ability of these matrices to neutralize peroxyl radicals [53].

Comparative evidence further supports the high antioxidant potential of acerola by-products. Acerola peels exhibited substantially higher antioxidant activity (approximately 4–7-fold) than strawberry residues across ABTS, DPPH, and FRAP assays, with consistent superiority also observed in ORAC measurements for both peels and seeds [2]. These differences are aligned with the higher concentrations of carotenoids, ascorbic acid, and phenolic compounds, including flavonoids, reported in acerola matrices [2,30,53], reinforcing the relationship between composition and antioxidant capacity. The data demonstrate that acerola peel shows promising potential as a source matrix for the extraction of phenolic compounds under different extraction conditions.

Methodological factors, particularly extraction techniques, further contribute to the variability observed across studies. Probe-type ultrasound-assisted extraction (UAE), for example, has been shown to enhance the recovery of phenolic compounds, leading to higher antioxidant activity in DPPH and FRAP assays under optimized conditions [60,61]. However, these responses are highly dependent on extraction parameters and sample characteristics, which limit direct comparisons between studies.

Despite the extensive characterization of acerola phytochemicals, important methodological limitations remain. Analytical approaches are often not standardized across studies, with variations in extraction solvents, detection techniques, and reporting units, limiting comparability and reproducibility. Furthermore, most studies report total phenolic or antioxidant capacity without detailed structural identification, hindering a precise understanding of compound-specific bioactivity. These limitations highlight the need for advanced analytical platforms and harmonized methodologies to improve the reliability and translational relevance of phytochemical data.

### 3.3. Foodomics Approaches to Acerola Composition and Functional Properties

Foodomics approaches, integrating metabolomics, transcriptomics, and proteomics, have contributed to advancing the understanding of the biochemical and molecular complexity of acerola and its by-products, providing complementary insights into the mechanisms underlying the biosynthesis of bioactive compounds associated with its functional properties [29,62,63]. Among these, metabolomics has been the most applied, allowing the characterization of low-molecular-weight compounds such as flavonoids, amino acids, organic acids, and lipids [62,63].

Untargeted analyses using advanced platforms (e.g., Ultra Performance Liquid Chromatography—Quadrupole Time-of-Flight Mass Spectrometry—UPLC-QTOF-MS) have shown clear variation in metabolite profiles across ripening stages, in agreement with the phytochemical composition described in Section 3.1 and Section 3.2. In particular, the consistent association between ascorbic acid content and antioxidant capacity highlights the role of vitamin C in the functional properties of acerola [63]. These findings reinforce the characterization of acerola as a phytochemically complex matrix whose composition is dynamically modulated during fruit development.

In addition to fresh pulp, metabolomic approaches have been successfully applied to acerola by-products, including pomace and seeds [63]. These matrices retain high concentrations of phenolic compounds such as quercetin, kaempferol, and isorhamnetin, supporting their potential as sustainable sources of bioactive ingredients for nutraceutical and functional food applications [4,5,63]. Such findings are particularly relevant in the context of agro-industrial valorization and circular economy strategies [1].

Transcriptomic and genomic approaches have provided important insights into the regulation of metabolic pathways associated with fruit composition. Gene expression studies in acerola have identified key enzymes involved in the L-galactose pathway of ascorbic acid biosynthesis, with transcriptional activity closely associated with vitamin C accumulation during fruit development [64,65]. Transcriptomic analyses further demonstrate coordinated regulation of genes involved in ascorbate metabolism, ethylene signaling, and secondary metabolism, highlighting the complexity of regulatory networks involving fruit ripening [65]. These findings reinforce that the marked variability in phytochemical composition observed among acerola cultivars is strongly influenced by genetic background and gene expression dynamics [31].

Despite these advances, several limitations still restrict the application of omics approaches in acerola research. Transcriptomic analyses are restricted to fresh tissues, as RNA is unstable and prone to rapid degradation, requiring rapid processing and stabilization to preserve transcript integrity [29,31]. In fruit systems, transcriptomic analysis has therefore been predominantly conducted in fresh or minimally processed tissues, particularly in investigations of fruit development and ripening dynamics [66].

On the other hand, proteomic analyses face additional challenges, as fruit tissues typically contain low amounts of protein and high levels of sugars, organic acids, and phenolic compounds, which interfere with protein extraction and downstream analyses [67,68]. These limitations are documented in fruit proteomics, where sample preparation is considered a critical and often limiting step for reliable protein identification and quantification [69,70].

Overall, while metabolomics has been successfully applied to both acerola pulp and derived products, genomic and transcriptomic approaches remain limited in scope, and proteomics are still incipient. The lack of integrated multi-omics studies represents a critical gap in the literature. Future research combining these approaches is essential to achieve a systems-level understanding of acerola biology [29], thereby linking genetic regulation to metabolite accumulation and, ultimately, to the biological effects attributed to this species.

### 3.4. Biological Effects of Acerola and Its By-Products in Animal Models

#### 3.4.1. Antioxidant and Anti-Inflammatory Effects

Preclinical evidence from animal models of metabolic stress indicates that acerola and its derived matrices exert antioxidant and anti-inflammatory effects (Table 3). These responses are consistently reflected in markers of oxidative stress, including reductions in lipid peroxidation and modulation of inflammatory mediators across different experimental conditions [26,28].

In rats fed a high-fat diet, administration of acerola by-products (400 mg/kg) reduced lipid peroxidation, with plasma levels decreasing from 8.19 to 3.86 nmol/L, with consistent effects also observed in other tissues such as the colon and liver [71]. These findings may be partly associated with the bioactive composition of these matrices, particularly phenolic compounds, ascorbic acid, and carotenoids, which have been previously characterized [5,39,71]. In parallel, phenolic compounds remain bioaccessible after ingestion and have been detected along the enterohepatic axis, encompassing both luminal compartments (cecal content and feces) and target tissues such as the colon and liver, supporting their physiological relevance and indicating that they reach sites of action in vivo [71]. This provides a mechanistic basis for the systemic redox response observed under dyslipidemic conditions. However, because these by-products represent a complex matrix, the relative contribution of individual constituents remains difficult to establish with certainty, especially in view of the overlapping mechanisms through which antioxidant compounds may act [73].

At the tissue level, antioxidant responses have also been observed in adipose depots following administration of acerola by-products. In obese rats fed a high-fat diet supplemented with 1% of these matrices, catalase activity increased in subcutaneous adipose tissue, accompanied by a reduction in subcutaneous fat mass, without significant changes in overall body weight gain [73]. This profile is consistent with localized modulation of oxidative status in adipose tissue, which may influence lipid handling independent of whole-body energy balance. The absence of changes in body weight further indicates that these responses are not driven by global metabolic alterations, but rather reflect tissue-specific redox adaptations.

Evidence from experimental findings using purified polysaccharide fractions extracted from acerola fruit provides mechanistic insight into the responses in high-fat diet models [26]. In mice exposed to diet-induced metabolic stress, these fractions reduced circulating and hepatic levels of pro-inflammatory cytokines (TNF-α, IL-6 and IL-1β), while improving oxidative status through decreased lipid peroxidation and restoration of antioxidant enzyme activity [26]. These responses were associated with activation of the Nrf2 signaling pathway, including increased expression of heme oxygenase-1 (HO-1) and NAD(P)H:quinone oxidoreductase-1 (NQO-1), key components of cellular antioxidant defense [26]. Improvements in cellular energy metabolism were further supported by restoration of ATP content and increased activity of respiratory chain complexes, reinforcing the link between redox and mitochondrial homeostasis [26]. However, as these findings derive from a single experimental model, their generalizability remains limited.

In acute inflammatory settings, acerola-derived polysaccharides have also been shown to exhibit anti-inflammatory and antioxidant activity. An arabinan-rich pectic polysaccharide fraction extracted from acerola pulp and peel attenuated carrageenan-induced paw edema and mechanical allodynia, reduced pro-inflammatory mediators including TNF-α, IL-1β, and prostaglandin E2 levels, and increased IL-10 levels in inflamed paw tissue, indicating a shift toward an anti-inflammatory profile [24]. These responses were accompanied by restoration of glutathione levels and antioxidant enzyme activity, along with reduced lipid peroxidation at the site of inflammation, supporting coordinated modulation of inflammatory and redox pathways [70]. These effects may be partly related to structural features of these polysaccharides, as pectic polysaccharide structures have been associated with the regulation of inflammatory mediators and immune responses [74]. This profile suggests that acerola-derived polysaccharides may act through the combined regulation of oxidative stress and inflammatory signaling.

Protective effects are also observed in chemically induced liver injury models. In Wistar rats exposed to carbon tetrachloride (CCl_4_), a widely used hepatotoxic agent that induces lipid peroxidation through the generation of reactive species, administration of lyophilized acerola bagasse extract (7 and 14 mg phenolic compounds/kg, for 21 days) reduced serum aminotransferase activities and preserved hepatic histological architecture [25]. These effects were accompanied by increased superoxide dismutase activity and total antioxidant capacity, reflecting attenuation of oxidative stress associated with xenobiotic exposure [25]. This is consistent with the known mechanisms of CCl-induced hepatotoxicity. Extrapolation of these findings should be approached with caution, given the constraints of the experimental model and the specific composition of the extract used.

Available experimental evidence indicates that acerola-derived matrices modulate antioxidant and inflammatory pathways in multiple tissues, particularly under conditions of metabolic and inflammatory stress [30,48,54,72]. Reported effects include reductions in oxidative damage, modulation of inflammatory mediators and activation of endogenous antioxidant defence systems, supporting the involvement of acerola bioactives in redox-sensitive biological processes.

#### 3.4.2. Metabolic and Hepatic Effects

Acerola and its derived matrices influence hepatic lipid homeostasis in experimental models of metabolic dysfunction (Table 4). Evidence from diet-induced steatosis and dyslipidemia indicates that these matrices modulate lipid synthesis, mitochondrial bioenergetics, and the distribution of lipids between intestinal and hepatic compartments [26,30,54]. Such effects are related to the presence of bioactive compounds, particularly phenolic constituents, ascorbic acid and dietary fiber fractions found in acerola by-products, as well as polysaccharide-rich extracts evaluated in specific experimental models, which may contribute to modulating lipid metabolism and hepatic function.

Studies conducted in high-fat diet-fed rodents, a widely used model of metabolic dysfunction-associated steatotic liver disease (MASLD), formerly termed non-alcoholic fatty liver disease, support the effects attributed to acerola-derived bioactive compounds [3,50,69]. Polysaccharides isolated from acerola fruit attenuated hepatic lipid accumulation and improved steatosis assessed by histology, with reduced expression of lipogenic enzymes such as fatty acid synthase (FAS), acetyl-CoA carboxylase (ACC) and stearoyl-CoA desaturase-1 (SCD-1) [1]. In parallel, mitochondrial adaptations were observed, including restoration of hepatic ATP content, increased activity of respiratory chain complexes I, IV and V, reduced expression of uncoupling protein 2 (UCP2), and stimulation of peroxisome proliferator-activated receptor gamma coactivator-1α (PGC-1α), supporting enhanced mitochondrial β-oxidation [1]. The extent of these changes is noteworthy, as they were obtained using a pectic polysaccharide fraction. The simultaneous modulation of lipogenesis and mitochondrial function suggests broader metabolic effects than would be expected from a single structural component, supporting a more active role of these compounds in hepatic metabolism [1,75].

Complementary evidence from whole acerola matrices further supports these metabolic effects. Orogastric administration of acerola by-products (400 mg/kg for 28 days) in high-fat diet-fed rats reduced body weight gain, serum lipid concentrations, blood glucose and hepatic fat accumulation, while improving insulin tolerance and increasing fecal bile acid excretion [30], consistent with coordinated changes in intestinal and hepatic lipid metabolism under diet-induced metabolic stress [30,49]. From a physiological perspective, the use of a complex matrix reflects the physiological context of food consumption, and the observed responses likely involve the integration of intestinal and hepatic processes, rather than being attributable to a single pathway, particularly given the overlap between lipid metabolism and bile acid turnover [54,71]. This pattern is consistent with the heterogeneous composition of acerola by-products, including fiber-rich fractions and oligosaccharides, along with a range of micronutrients and bioactive compounds such as carotenoids, ascorbic acid, organic acids, and phenolic constituents, which may collectively contribute to their modulatory effects on lipid metabolism, as characterized in the original study [30].

Comparable responses were observed in a dyslipidemia-driven model, where supplementation with an acerola processing by-product (400 mg/kg, orogastric administration for 28 days) reduced visceral and hepatic fat deposition and improved circulating lipid parameters in female Wistar rats [54]. Increased fecal fat excretion and alterations in organic acid concentrations suggest that intestinal processes contribute to reduced hepatic lipid burden. Under the same conditions, by-products from cashew and guava were also evaluated, although the effects were more pronounced in the acerola group, likely reflecting differences in matrix composition, indicating that these responses may depend on the specific bioactive profile of each matrix rather than representing a generalizable effect across fruit by-products [54]. Histological evaluation further supported attenuation of diet-induced hepatic fat accumulation [54].

Acerola processing residues also influenced basal lipid homeostasis under non-pathological conditions. In healthy Wistar rats, supplementation (400 mg/kg, orogastric administration for 28 days) was associated with reduced serum lipid concentrations and increased hepatic retinol deposition without adverse effects on body composition [52]. By-products from cashew and guava were also evaluated, with partially overlapping but distinct metabolic responses across three matrices. While acerola and guava by-products were associated with reductions in serum lipid levels, cashew by-products showed a more pronounced effect on blood glucose [52]. The persistence of these responses in healthy animals indicates that the effects of acerola-derived materials are not restricted to dyslipidemic states, but may also reflect modulation of basal lipid metabolism and vitamin A homeostasis. These differences may be partly explained by variations in the compositional profiles of the matrices [39,54], particularly the higher ascorbic acid content observed in acerola by-products, alongside comparable carotenoid levels across the three fruits, which may contribute to differences in their metabolic effects.

Acerola-derived matrices modulate hepatic lipid metabolism in experimental models, with reported effects on hepatic lipid accumulation, circulating lipid parameters and pathways related to mitochondrial and enterohepatic regulation. Available data indicate a consistent influence on mechanisms involved in lipid metabolism and metabolic homeostasis under conditions of metabolic dysfunction [30,54,71,72].

#### 3.4.3. Other Biological Effects

Acerola and its derived matrices are also explored in experimental contexts beyond antioxidant and metabolic responses. Preclinical studies describe gastroprotective effects, attenuation of genotoxic damage, and improvements in fatigue-related performance, alongside modulation of neuroenergetic parameters under dietary or physical stress conditions [75,76,77]. These effects are observed across diverse experimental settings; however, variations in study design and outcome measures limit direct comparisons between studies (Table 5).

Gastrointestinal effects have been observed in mice models of ethanol-induced gastric injury. Water-soluble polysaccharides extracted from acerola processing residues preserved gastric mucosal architecture and attenuated oxidative damage in vivo [77]. Pretreatment (1 mg/kg, orogastric administration) prevented ethanol-induced depletion of gastric glutathione and reduced malondialdehyde levels in stomach tissue, indicating decreased lipid peroxidation and maintenance of mucosal redox balance. The lack of cytotoxicity effects in intestinal epithelial cells further supports the tolerability of these fractions within the experimental setting evaluated [77]. Comparable gastroprotective responses have also been reported for polysaccharide fractions derived from other fruit by-products [3,77], including cashew and pineapple, whereas similar effects were not observed for mango and passion fruit, highlighting variability in bioactivity among matrices. These differences are consistent with variations in monosaccharide composition, with acerola polysaccharides enriched in arabinose, while mango and passion fruit fractions are predominantly glucose-based [77], which may influence their structural properties and interaction with the gastric mucosa [79]. Given the acute nature of the model, responses likely reflect short-term protection against chemically induced stress.

Genoprotective responses further extend the range of biological effects attributed to acerola [78,80,81]. Exposure to dietary metals may include both essential and toxic elements capable of inducing DNA damage, either directly or through the generation of reactive oxygen species: a process associated with the development of several diseases [80]. Acerola contains a range of antioxidant compounds that may contribute to mitigating metal-induced oxidative stress and subsequent DNA damage [81]. In mice exposed to iron-induced genotoxicity, pre-treatment with acerola juice, regardless of maturation stage, reduced the frequency of micronuclei in bone marrow cells, indicating antimutagenic activity as assessed by the micronucleus assay [78]. This response is consistent with a protective effect against oxidative damage to the genetic material, suggesting that acerola-derived antioxidants may interfere with early events involved in metal-induced genotoxicity [80]. The absence of marked differences between unripe and ripe juice also indicates that this effect may be preserved across maturation stages, despite expected variations in fruit composition. Although these findings support a protective role against oxidative DNA damage, the use of a single genotoxic agent and acute exposure conditions limits the generalization of these effects across different types of genomic stress.

Neuroenergetic alterations also emerge in the context of diet-related metabolic disturbances. Using a cafeteria diet model of obesity, acerola juice intake partially restored key enzymes involved in cerebral energy metabolism, including citrate synthase and mitochondrial respiratory complexes, across metabolically relevant regions such as the hypothalamus and prefrontal cortex [76]. Industrial acerola juice reversed diet-induced inhibition of hypothalamic complex I activity, whereas unripe and ripe preparations restored citrate synthase levels in a region-specific manner, with differential effects on mitochondrial function rather than a global increase in bioenergetic activity [76]. Not all respiratory alterations were normalized, reinforcing the notion of targeted mitochondrial adaptation. This selective modulation of the response suggests a partial restoration of cerebral energy homeostasis, potentially contributing to the maintenance of neuronal function under metabolic stress [76,78], which may be influenced by variation in phytochemical composition across juice preparations.

Fatigue during sustained physical effort provides a useful framework for examining bioenergetic adaptations. A weight-loaded swimming model was used to assess an arabinan-rich pectic polysaccharide isolated from acerola pulp after 28 days of oral supplementation (50–200 mg/kg), resulting in a prolonged time to exhaustion [75]. Treated groups showed higher post-exercise plasma glucose and triglyceride concentrations, together with increased lactate levels, indicating a shift in carbohydrate and lipid mobilization rather than a reduction in metabolic demand [75]. At higher doses, mitochondrial respiratory capacity in permeabilized skeletal muscle fibers increased following adenosine diphosphate (ADP) stimulation, consistent with enhanced oxidative phosphorylation under high-energy requirements [75]. Glutathione levels were higher in the hippocampus, whereas lipid peroxidation and plasma markers of hepatic injury remained unchanged [75]. The dose-dependent nature of these responses, particularly the enhancement of mitochondrial respiratory capacity at higher doses, suggests that the metabolic effects of the arabinan-rich pectic polysaccharide are modulated by the level of supplementation, reflecting a threshold for bioenergetic adaptation under conditions of increased energy demand [75]. A coordinated pattern of peripheral and central adaptations emerges, with improved mitochondrial function in skeletal muscle and selective modulation of antioxidant status in the brain, supporting enhanced tolerance to metabolic stress during sustained exercise.

Acerola-derived matrices exert biological effects in experimental models beyond antioxidant and metabolic regulation, including gastrointestinal protection, modulation of genomic stability, neuroenergetic responses and improved fatigue-related performance [49,75,77,78]. Together, these observations indicate that the biological activity of acerola extends beyond a single physiological domain and involves multiple organ systems.

### 3.5. Evidence from Human Studies

Human studies evaluating the biological effects of acerola remain more limited than preclinical investigations and are mainly based on small-scale interventions. Available trials point to antioxidant-related effects, although results are not consistent between markers [24,25]. More recently, clinical investigations have explored additional physiological outcomes beyond global antioxidant capacity.

In a pilot trial involving 22 elite endurance athletes supplemented with 300 g/day of acerola pulp for three weeks, Vítek et al. [24] reported reductions in circulating immunoglobulins (including IgG subclasses and IgA), complement component C1q, and selected metabolic markers, whereas total antioxidant status and glutathione reductase activity remained unchanged. Phytochemical profiling of the pulp showed high vitamin C content and flavonoids such as quercitrin and aceronidin, which may be related to the biological effects reported [24]. The use of a highly trained population, together with the short intervention period and limited sample size, should be considered when interpreting these findings, as both baseline metabolic status and exercise-related adaptations may influence the outcomes.

The lack of variation in global redox indices may also be related to the limited specificity of broad antioxidant assays, particularly in individuals with already optimized redox homeostasis. The inclusion of more specific markers of oxidative damage, such as lipid peroxidation or protein oxidation products, together with quantitative cytokine profiling, could provide a clearer assessment of whether acerola directly influences redox balance or is more closely associated with inflammatory and metabolic adjustments. Larger placebo-controlled trials with longer follow-up periods are needed to strengthen causal interpretation and improve the clinical relevance of these observations.

Earlier pharmacokinetic evidence by Uchida et al. [25] demonstrated that ingestion of acerola juice containing 50 mg of ascorbic acid resulted in greater plasma exposure and lower urinary excretion compared with isolated ascorbic acid, supporting a matrix-dependent modulation of vitamin C bioavailability. However, the acute design and the absence of functional clinical endpoints limit inference regarding sustained physiological effects.

Most studies rely on fresh pulp or juice, whereas evidence on acerola by-products in human populations remains limited, despite their well-characterized phytochemical profile. This gap underscores the need for clinical trials using standardized by-product matrices and incorporating redox, inflammatory, and metabolic endpoints, allowing a clearer link between compositional data and clinically relevant outcomes.

A key limitation in current clinical research using acerola and its derived matrices as an intervention is the lack of standardized intervention protocols, particularly regarding dose, duration, and matrix composition. In addition, the absence of human studies specifically evaluating acerola by-products restricts translational interpretation, especially given their high phytochemical density and potential industrial application. Addressing these limitations will be essential to establishing the functional and clinical relevance of acerola-derived bioactives.

## 4. Critical Appraisal and Translational Considerations

Variability in the phytochemical composition of acerola remains a key challenge for interpreting its biological effects. Differences related to cultivars, fruit maturity, processing conditions, and the use of distinct matrices (e.g., fresh pulp, juice, by-products, extracts and isolated compounds) result in heterogeneous profiles of bioactive compounds, including ascorbic acid, polyphenols, and dietary fiber. This heterogeneity limits comparability across studies and complicates the interpretation of matrix-dependent effects. The lack of standardization in analytical methods used for compound identification and quantification further contributes to this limitation.

From a precision health perspective, foodomics and multi-omics approaches may provide an integrative framework for linking the characterization of acerola-derived matrices to their biological effects. In acerola and its by-products, high-throughput platforms such as metabolomics and proteomics may improve the profiling of bioactive compounds and their transformations during processing, thereby refining the characterization of phytochemical composition and functional potential, as discussed in Section 3.3. At the molecular and cellular levels, transcriptomics, proteomics, and metabolomics have supported the identification of molecular targets, signaling pathways, and gene–nutrient interactions involved in the response to these phytochemical matrices [29,82]. Integrative analyses may further clarify modulation of antioxidant and cytoprotective pathways, such as Nrf2/Keap1, as well as inflammatory pathways including NF-κB, although their application in acerola research remains limited [83].

These approaches may also help elucidate interactions between acerola bioactives and the gut microbiota, including shifts in microbial composition and metabolite production (e.g., short-chain fatty acids), which are recognized mediators of systemic effects [84,85,86]. Nevertheless, current evidence remains limited and largely indirect, and the specific contribution of acerola-derived compounds to microbiota-mediated responses has not yet been clearly established. The integration of omics data from both food matrices and biological responses may additionally support the identification of biomarkers related to efficacy, interindividual variability, and metabolic responsiveness; however, the validation of such markers in the context of acerola intake remains scarce [87].

In parallel, micro- and nanotechnology-based delivery systems are being explored to improve the stability and bioavailability of acerola-derived bioactives. Techniques such as nanoencapsulation and nanocarriers may protect labile compounds, including ascorbic acid and polyphenols, from degradation, enhance intestinal absorption, and allow controlled release and tissue-specific distribution [88,89]. Despite these advances, most evidence is derived from experimental systems, and their applicability in human settings remains to be demonstrated. The combination of mechanistic insights from omics approaches with advanced delivery systems may represent a promising direction for future research, although its translational potential requires further validation in well-controlled studies [90,91].

**Figure 3:**
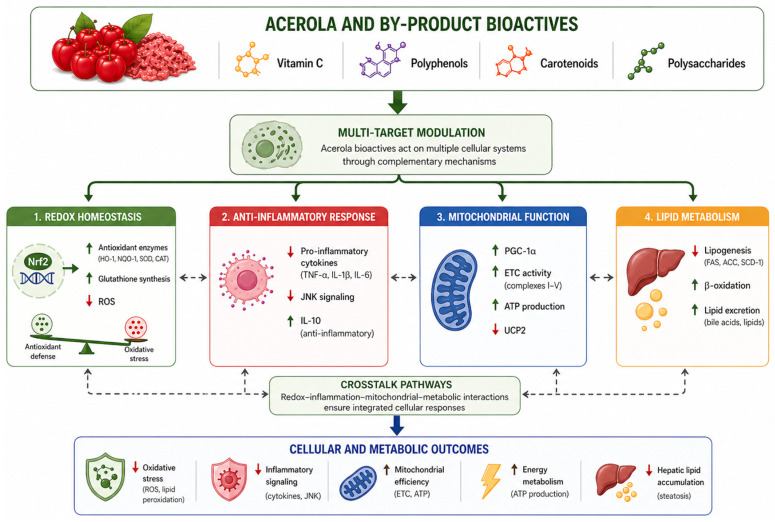
Molecular mechanisms underlying the biological effects of acerola and by-product bioactives, HO-1, Heme oxygenase-1; NQO-1, Quinone oxidoreductase-1; SOD, Superoxide dismutase; CAT, Catalase; ROS, Reactive oxygen species; PGC-1α, Peroxisome proliferator-activated receptor gamma coactivator-1α; ATP, Adenosine Triphosphate; UCP2, Uncoupling protein 2; TNF-α, Tumor necrosis factor alpha; IL-1β, Interleukin-1 beta; IL-6, Interleukin-6; JNK, Jun N-terminal kinases; IL-10, Interleukin-10, FAS, Fatty acid synthase; ACC, Acetyl-CoA carboxylase; SCD-1, Stearoyl-CoA desaturase-1; upward arrows (↑), indicates increase; downward arrows (↓), indicates decrease.

Although the biological effects of acerola and its by-products are suggested by relatively consistent experimental evidence [30,44,52,54,71,72], important limitations remain. Uncertainties regarding bioavailability, incomplete characterization of bioactive fractions, and inconsistent dosage strategies limit the establishment of clear relationships between intake and systemic effects. A summary of the main biological effects reported for acerola pulp and its by-products in experimental and clinical settings is presented in Figure 3.

Most preclinical studies have been conducted in high-fat diet models of dyslipidemia and obesity [30,44,52,54,71,72]. Although these models are relevant for cardiometabolic research and frequently report antioxidant and metabolic effects, particularly along the enterohepatic axis, their predominance restricts broader pathophysiological interpretation. Evidence in other contexts, including cancer, microbiota-mediated responses, and neurodegenerative processes, remains relatively scarce, limiting the generalization of these findings across biological systems. Expanding experimental designs to include a wider range of biological conditions may help determine whether the observed effects are model-specific or reflect more general physiological responses.

Comparative studies between acerola pulp and its by-products remain scarce, particularly those evaluating whole matrices alongside isolated bioactive fractions. This distinction is relevant because differences in phytochemical density, fiber content, and matrix complexity may influence bioavailability and biological responses. Future studies may benefit from head-to-head designs across systems, animal models, and human trials to clarify relative efficacy, dose equivalence, and underlying mechanisms.

Among tropical fruit residues, acerola by-products appear to be supported by a relatively more consistent body of preclinical evidence. By-products from fruits such as cajá (*Spondias mombin* L.), umbu (*Spondias tuberosa* Arruda), guava (*Psidium guajava*), and cashew apple (*Anacardium occidentale*) have shown antioxidant and metabolic potential [54,92,93,94]; however, most available studies remain restricted to compositional analyses or assays, with fewer investigations addressing systemic and tissue-level effects in vivo. This suggests that acerola has been more extensively explored in experimental settings, although clinical validation remains limited. While animal studies provide important mechanistic insights, their predominance, particularly in models of metabolic disorders, limits broader translational applicability. Greater standardization of study design and increased inclusion of human studies will be necessary to establish efficacy, safety, and optimal intake conditions.

The valorization of acerola by-products is increasingly aligned with sustainability and bioeconomy strategies. The recovery and use of pomace, peels, and seeds may help reduce agro-industrial waste while generating value-added ingredients for food, nutraceutical, and pharmaceutical applications. These practices are consistent with circular economy principles and may support the United Nations Sustainable Development Goals (SDGs), particularly those related to responsible production (SDG 12), innovation (SDG 9), and health (SDG 3). Given Brazil’s leading role in acerola production, the use of these by-products may also contribute to regional development, especially in semi-arid areas, while encouraging the development of standardized bioactive ingredients. In this context, acerola by-products represent promising resources within more sustainable production systems rather than as organic waste.

This review has inherent limitations that should be acknowledged. Although based on a structured search, its narrative design does not allow for quantitative synthesis and may introduce selection bias. In addition, the heterogeneity of the available evidence and predominance of preclinical studies limit comparability and translational interpretation.

## 5. Conclusions and Future Perspectives

Acerola and its by-products constitute phytochemically complex matrices rich in ascorbic acid, hydroxycinnamic acids, flavonoids, carotenoids, and bioactive polysaccharides. Preclinical evidence suggests that these constituents may modulate redox homeostasis and inflammatory signaling pathways, potentially through coordinated antioxidant effects and the regulation of lipid and glucose metabolism. By integrating current evidence across compositional, experimental, and emerging translational approaches, this review summarizes the current understanding of acerola-derived matrices as sources of bioactive compounds. However, most of the available evidence derives from experimental models and is limited by variability in study design, lack of standardization, and the scarcity of direct comparative studies, which restricts translational interpretation.

Future studies should prioritize detailed phytochemical characterization, dose–response relationships, bioavailability assessment, and well-controlled clinical trials incorporating molecular and mechanistic biomarkers to better clarify efficacy and safety.

Current evidence suggests the promising but still preliminary biological relevance of acerola-derived matrices. Further advances in standardization and translational validation will be necessary to determine their applicability in technological and clinical contexts.

## Figures and Tables

**Table 2:** Proximate composition and main bioactive compounds of acerola by-products.

Parameter (g/100 g)	Mean Value	By-Product	Reference
Moisture	77.0	Mixture of lumps, seeds and peels	[17]
Ash	2.8	Mixture of lumps, seeds and peels	[17]
Proteins	8.3	Mixture of lumps, seeds and peels	[17]
Lipids	2.45	Mixture of peels and seeds	[54]
Carbohydrates	52.0	Mixture of lumps, seeds and peels	[17]
Fibers	34.2	Mixture of lumps, seeds and peels	[17]
Bioactive compounds			
Anthocyanins (mg/100 g)	42.30	Mixture of lumps, seeds and peels	[17]
Chlorophyll (µg/mL)			
Chlorophyll a	5.49	Green residue ‡	[51]
Chlorophyll b	4.06		
Total chlorophyll	9.56		
β-carotene (mg/g)	5.12	Peel	[2]
	3.24	Seed	
Total carotenoids (mg/100 g)	4.99	Mixture of peels, residual pulp, and seed	[30]
	0.263 *	Mixture of peels, residual pulp, and seeds	[52]
	0.070 *	Green residue ‡	[51]
Ascorbic acid (mg/100 g)	1063.50	Mixture of lumps, seeds and peels	[17]
	2898.83	Mixture of peels, residual pulp, and seeds	[30]
	1012.19	Green residue ‡	[51]
	202.21 *	Mixture of peels, residual pulp, and seeds	[52]
Organic acids (mg/g)			
Formic acid	7.31	Mixture of peels, residual pulp, and seeds	[30]
Malic acid	6.60		
Succinic acid	15.46		
Tannins (mg/g)			
Condensed	3.86	Peel	[2]
Hydrolyzable	28.21	Peel	
Condensed	2.90	Seed	
Hydrolyzable	19.76	Seed	
Phenolic acids (µg/g)			
Ellagic acid	50.40	Mixture of peels, residual pulp, and seeds	[30]
2-Hydroxybenzoic	1374.80		
3,4,5-Trihydroxybenzoic acid	1.51		
3,5-Dimethoxy-4-hydroxybenzoic acid	101.60		
Caftaric acid	86.58		
4-Hydroxybenzoic acid	789.60		
Cinnamic acid	25.60		
3,4-Dihydroxycinnamic acid	7.35		
3-O-Caffeoylquinic acid	14.67		
4-Hydroxycinnamic acid	65.50		
4-Hydroxy-3-methoxycinnamic acid	51.97		
4-Hydroxy-3,5-dimethoxycinnamic acid	137.60		
Flavonoids (μg/g)			
Procyanidin A2	2.38	Mixture of peels, residual pulp, and seeds	[30]
Procyanidin B1	7.26		
Procyanidin B2	43.33		
Epigallocatechin gallate	4.48		
Epicatechin gallate	3.37		
Catechin	1622.50		
Kaempferol 3-glicoside	88.37		
Rutin	22.46		
Quercetin	250.80		
Quercetin-3-glycoside	13.39		
Myricetin	333.80		
Hesperedin	29.54		
Hesperetin	13.00		
Naringenin	48.90		
Chrysine	8.40		
Stilbenes (μg/g)			
Trans-resveratrol	6.19	Mixture of peels, residual pulp, and seeds	[30]
Cis-resveratrol	15.67		
Total phenolic compounds (mg/100 g)			
	1155.20 †	Mixture of lumps, seeds and peels	[17]
	10.680 *	Mixture of seeds and peels	[54]
	536.64 *	Mixture of peels, residual pulp, and seeds	[30]
	7276.25 * †	Green residue ‡	[51]
Minerals (mg/g)			
Potassium	58.32	Mixture of peels, residual pulp, and seeds	[30]
Calcium	31.91		
Magnesium	2.82		
Chlorine	2.25		
Phosphorus	2.43		
Sulfur	1.20		
Iron	0.25		
Zinc	0.09		
Copper	0.05		

Values are presented as means to ensure consistency across studies, as standard deviations were not consistently reported in all original sources. * Indicates data that were standardized in mg/100 g to improve comparability of the results. † Indicates data reported as mg GAE/100 g in the original study. ‡ Green residue is the term used in the original study to describe acerola processing by-products; its specific composition (e.g., peel, seed, or pulp fractions) was not detailed by the authors.

**Table 3:** Antioxidant and anti-inflammatory effects of acerola-derived matrices in animal models.

Matrix/Preparation	Dose, Route and Duration	Animal Model (*n*)	Experimental Condition	Main Outcomes	Reference
Acerola by-products (peels, residual pulp, and seeds; freeze-dried)	400 mg/kg, orogastric, 28 days	Wistar rats (*n* = 8/group)	HFD-induced dyslipidemia	↓ Lipid peroxidation; ↑ TAC (plasma, colon, liver)	[71]
Acerola by-products	Dietary (diet incorporation, 1%), 4 weeks	Wistar rats (*n* = 6/group)	Diet-induced obesity	↑ CAT (subcutaneous adipose tissue)	[72]
Acerola polysaccharides (isolated fraction)	200–800 mg/kg, oral, 9 weeks	C57BL/6 mice (*n* = 6/group)	HFD-induced MASLD (reported as NAFLD in the original study)	↓ TNF-α, IL-6, IL-1β; ↑ SOD, CAT; activation of Nrf2 pathway	[26]
Acerola polysaccharides (arabinan-rich fraction)	0.1–1 mg/kg, intraperitoneal	Swiss mice (*n* = 4–8/group)	Carrageenan-induced inflammation	↓ TNF-α, IL-1β, PGE2; ↑ GSH, SOD, CAT	[23]
Lyophilized acerola bagasse extract	7–14 mg phenolics/kg, orogastric, 21 days	Wistar rats (*n* = 6/group)	CCl_4_-induced liver injury	↓ ALT, AST; ↓ MDA; ↑ SOD, TAC	[25]

Abbreviations: ALT, alanine aminotransferase; AST, aspartate aminotransferase; CAT, catalase; CCl_4_, carbon tetrachloride; GSH, reduced glutathione ratio; HFD, high-fat diet; IL-1β, interleukin-1 beta; MASLD, metabolic dysfunction-associated steatotic liver disease; MDA, malondialdehyde; Nrf2, nuclear factor (erythroid-derived-2)-like 2; SOD, superoxide dismutase; PGE_2_, prostaglandin E2; TAC, total antioxidant capacity; TNF-α, tumor necrosis factor alpha; upward arrows (↑) = indicates increase; downward arrows (↓) = indicates decrease.

**Table 4:** Metabolic and hepatic effects of acerola-derived matrices in experimental models.

Matrix/Preparation	Dose, Route and Duration	Animal Model (*n*)	Experimental Condition	Main Outcomes	Reference
Acerola-derived polysaccharides (isolated fraction)	200–800 mg/kg, oral, 9 weeks	C57BL/6 mice (*n* = 6/group)	HFD-induced MASLD (reported as NAFLD in the original study)	↓ Hepatic lipid accumulation; ↓ SREBP-1c, FAS, ACC, SCD-1; ↓ UCP2; ↑ mitochondrial complexes I, IV, V; ↑ PGC-1α	[26]
Acerola by-products (peels, residual pulp, and seeds; freeze-dried)	400 mg/kg, orogastric, 28 days	Wistar rats (*n* = 8/group)	HFD-induced metabolic dysfunction	↓ Liver fat accumulation; ↓ serum lipids; ↓ blood glucose; ↑ insulin tolerance; ↑ fecal bile acid excretion	[30]
Acerola by-products (peels, residual pulp, and seeds; freeze-dried)	400 mg/kg, orogastric, 28 days	Wistar rats (*n* = 8/group)	Diet-induced dyslipidemia	↓ Hepatic fat deposition; ↓ serum lipids; ↓ visceral fat; ↑ fecal fat excretion; ↑ organic acids	[54]
Acerola by-products (peels, residual pulp, and seeds; freeze-dried)	400 mg/kg, orogastric, 28 days	Wistar rats (*n* = 8/group)	Healthy condition	↑ Hepatic retinol deposition; ↓ serum lipids	[52]

Abbreviations: FAS, fatty acid synthase; HFD, high-fat diet; NAFLD, non-alcoholic fatty liver disease; PGC-1α, peroxisome proliferator-activated receptor gamma coactivator-1α; SCD-1, stearoyl-CoA desaturase-1; SREBP-1c, sterol regulatory element-binding protein-1c; UCP2, uncoupling protein 2; upward arrows (↑) = indicates increase; downward arrows (↓) = indicates decrease.

**Table 5:** Other biological effects of acerola-derived matrices in experimental models.

Matrix/Preparation	Dose, Route and Duration	Animal Model (*n*)	Experimental Condition	Main Outcomes	Reference
Acerola-derived polysaccharides (pectic fraction)	50–200 mg/kg, orogastric, 28 days	Swiss mice (*n* = 6/group)	Weight-loaded swimming model	↑ Time to exhaustion; ↑ mitochondrial respiration (skeletal muscle); ↑ hippocampal GSH	[75]
Polysaccharides from acerola by-products (peels, residual pulp, and seeds)	1 mg/kg, orogastric, 1 day	Wistar rats (*n* = 6/group)	Ethanol-induced gastric injury	↑ Gastric GSH; ↓ MDA (stomach); preservation of gastric mucosa	[77]
Acerola juice (unripe and ripe)	0.1 mL/10 g, orogastric, 1 day	CF1 mice (*n* = 10/group)	Genotoxicity induced by iron	↓ micronucleated erythrocytes (bone marrow)	[78]
Acerola juice (unripe, ripe, industrial)	0.1 mL/10 g, orogastric, 4 weeks	Swiss mice (*n* = 6/group)	Cafeteria diet-induced obesity	Restoration of citrate synthase activity; partial recovery of mitochondrial complex I activity (hypothalamus)	[76]

Abbreviations: GSH, reduced glutathione; MDA, malondialdehyde; upward arrows (↑) = indicates increase; downward arrows (↓) = indicates decrease.

## Data Availability

No new data were created or analyzed in this study. Data sharing is not applicable to this article.

## Abbreviations

ADP	Adenosine diphosphate
CAT	Catalase
DNA	Deoxyribonucleic acid
FAS	Fatty acid synthase
FRAP	Ferric reducing antioxidant power
GSH	Glutathione
GSSG	Oxidized glutathione
HFD	High-fat diet
HO-1	Heme oxygenase-1
IL-1β	Interleukin-1 beta
IL-10	Interleukin-10
JNK	Jun N-terminal kinases
MASLD	Metabolic dysfunction-associated steatotic liver disease
MDA	Malondialdehyde
ORAC	Oxygen radical absorbance capacity
Nrf2	Nuclear factor (erythroid-derived-2)-like 2
NQO-1	NAD(P)H:quinone oxidoreductase-1
PGC-1α	Peroxisome proliferator-activated receptor gamma coactivator-1α
PGE_2_	Prostaglandin E2
SCD-1	Stearoyl-CoA desaturase-1
SOD	Superoxide dismutase
SREBP-1c	Sterol regulatory element-binding protein-1c
TAC	Total antioxidant capacity
TNF-α	Tumor necrosis factor alpha
UCP2	Uncoupling protein 2
